# Dengue and severe dengue with neurological complications: a challenge for prevention and control

**DOI:** 10.1055/s-0044-1792091

**Published:** 2024-12-03

**Authors:** Emanuelle de Oliveira Francelino, Marzia Puccioni-Sohler

**Affiliations:** 1 Universidade Federal do Estado do Rio de Janeiro, Escola de Medicina e Cirurgia, Rio de Janeiro RJ, Brazil.; 2 Universidade Federal do Rio de Janeiro, Faculdade de Medicina, Programa de Pós-Graduação em Doenças Infecciosas e Parasitárias, Rio de Janeiro RJ, Brazil.

**Keywords:** Dengue, Disease Prevention, Vaccines, Vector Control of Diseases, Insect Repellents, Dengue, Prevenção de Doenças, Vacinas, Controle de Vetores de Doenças, Repelentes de Insetos

## Abstract

Dengue is the main urban arbovirus in the Americas. The disease manifests in a varied spectrum: from asymptomatic cases to those with neurological involvement, which is considered a severe form of the disease. Its annual reemergence represents a serious public health problem. The rise in the number of cases causes an increase in the number of patients with neurological manifestations of the disease, which can range from headaches to more serious conditions such as encephalitis and Guillain-Barré syndrome, with high potential of death or sequelae. Dengue prevention and control strategies should also be a concern for neurologists. The aim of the present study is to carry out a narrative review of the current methods to prevent dengue fever and its severe forms, such as cases with neurological complications. The main control measures include vaccination, which is still carried out on a small scale, vector control, and individual protection. The CYD-TDV/Dengvaxia and TAK-003/DENVax vaccines, licensed for use by the Brazilian National Health Regulatory Agency (Agência Nacional de Vigilância Sanitária, ANVISA, in Portuguese), show efficacy against hospitalizations of 72.7% (95% confidence interval [95%CI]: 62.3–80.3%) and of 90.4% (95%CI: 82.6–94.7%) respectively. The TV003/TV005 vaccine, which is being studied by Intituto Butantan in Brazil, shows promising results, with an efficacy of 79.6% for symptomatic dengue. Vector control is based on biotechnological and behavioral measures, as well as on the improvement of basic sanitation conditions. The main individual protection measure is the use of topical repellents (icaridin). All of these actions represent important tools for the prevention of dengue fever and its neurological complications.

## Resumo

A dengue é a principal arbovirose urbana das Américas. A doença manifesta-se num espectro variado: desde casos assintomáticos até aqueles com envolvimento neurológico, que são considerados formas graves da doença. Sua reemergência anual representa um grave problema de saúde pública. O aumento do número de casos provoca o aumento do número de pacientes com manifestações neurológicas da doença, que podem variar de cefaleia a condições graves, como a encefalite e a síndrome de Guillain-Barré, com elevado potencial de morte ou sequelas. As estratégias de prevenção e controle da dengue também devem ser uma preocupação dos neurologistas. O objetivo deste estudo é fazer uma revisão narrativa dos métodos atuais de prevenção da dengue e das formas graves, como os casos com complicações neurológicas. As principais medidas de controle incluem a vacinação, que ainda é realizada em pequena escala, o controle do vetor e a proteção individual. As vacinas CYD-TDV/Dengvaxia e TAK-003/DENVax, aprovadas para uso pela Agência Nacional de Vigilância Sanitária (Anvisa), apresentam eficácia contra hospitalizações de 72,7% (intervalo de confiança de 95% [IC95%]: 62,3–80,3%) e de 90,4% (IC95%: 82,6–94,7%), respectivamente. A vacina TV003/TV005, que está sendo desenvolvida pelo Instituto Butantan no Brasil, apresenta resultados promissores, com uma eficácia de 79,6% para a dengue sintomática. O controle vetorial baseia-se em medidas biotecnológicas e comportamentais, assim como na melhoria das condições de saneamento básico. A principal proteção individual é o uso de repelentes tópicos (icaridina). Todas essas ações representam importantes ferramentas para a prevenção da dengue e das suas complicações neurológicas.

## INTRODUCTION

Infectious diseases are a major global concern, especially in tropical countries. Dengue fever is the most important arbovirus infecting humans, whose main transmitter is a female mosquito of the genus *Aedes* , ( *Ae. aegypti* and *Ae. albopictus* ). 1 2 The dengue virus (DENV) belongs to the *Flaviviridae* family and has 4 serotypes of importance in human infection: DENV-1, -2, -3, and -4. 1 Secondary exposure to a different serotype is the main risk factor for the emergence of serious conditions such as dengue hemorrhagic fever and dengue shock syndrome due to cross-reaction. 3

The global incidence of dengue has risen sharply over the last decades. The World Health Organization (WHO) recorded more than 5 million cases, with more than 5 thousand deaths in 80 countries/territories in 2023. Around 80% of these dengue cases were reported on the American continent, which has epidemics of the disease every three to five years. 3 In Brazil, there were 6,237,241 probable cases of dengue by the first semester of 2024, with an alarming incidence rate of 3,071 cases per 100 thousand inhabitants and 4,367 deaths. 4 This is the highest number of dengue cases ever recorded over 1 year in Brazil. The most prevalent serotypes in this period included DENV-1 and -2, but all 4 serotypes were found circulating in the population. 4

Dengue manifests itself in a varied spectrum of symptoms, ranging from a mild febrile illness to neurological involvement in severe cases. 1 5 These complications occur in 1 to 20% of diagnosed dengue cases. However, there is a strong suspicion of underreporting, since the neurological manifestations may occur even in patients with few or no other systemic symptoms of the disease. 6 7 The main neurological complications associated with dengue include encephalitis, hypoxic/metabolic encephalopathy, myelitis, Guillain-Barré syndrome, and hemorrhagic stroke in dengue hemorrhagic fever. 8 9

In view of the large contingent of individuals exposed to the virus, the broad spectrum of symptoms, and the possibility of severe cases with irreversible sequelae or death, this study aims to review the updated measures of dengue prevention and, thus, avoid serious complications, such as those affecting the nervous system.

## NEUROLOGICAL COMPLICATIONS

Neurological complications associated with DENV serotypes are manifested through four different mechanisms:

- Metabolic disorders: encephalopathy and hypokalemic paralysis;
- Direct viral invasion, in cases of encephalitis, meningitis and myelitis;
- Autoimmune reactions, such as Guillain Barré syndrome, optic neuritis and neuromyelitis, acute disseminated encephalomyelitis; and
- Stroke associated with dengue hemorrhagic fever. 8

The DENV-2 and -3 serotypes have the greatest neuroinvasive potential. 6 In the acute phase of the disease, consisting of the first 5 to 7 days, manifestations associated with metabolic and hemorrhagic disorders and direct invasion of the virus into the nervous system are the most common; while in the subacute-chronic phase, after 7 to 15 days following the first days of symptoms, autoimmune manifestations are the most recurrent. 7

There are studies aimed at pharmacological treatments for the neurological complications of dengue, such as the tanreqing (TRQ) injection. It was approved by the Chinese Food and Drug Administration and used to treat inflammation in the brain and other related diseases (acute cholecystitis, and infantile acute pneumonia). This appears to inhibit the DENV encephalitis by suppressing the activation of the NLRP3 inflammasome, which are a group of cytoplasmic receptors that recognize microorganisms (bacteria, viruses, parasites...), responsible for neuroinflammation and neuronal loss. Inflammasome activation is observed in microglia and other resident cells of the central nervous system. The use of TRQ in DENV encephalitis has demonstrated protective effects by reducing microglial activation and, consequently, the inflammatory response associated with DENV-2 in vitro and in vivo. 10 However, this and other studies are still in the experimental phase, with prevention being the most promising way to avoid the complications of dengue. 10

## DENGUE PREVENTION

### Vaccines

The main challenges in developing vaccines are to cover the four serotypes of the DENV and to prevent cross-immunity, which consists of more serious conditions in a postvaccination infection in patients who are seronegative for one of the serotypes less covered by the vaccine administered. There are currently at least seven vaccines in different stages of development and clinical trials. 11

The first vaccine to be licensed for use in Brazil on the basis of the three phases of clinical trials and approved for use in several countries was CYD-TDV, also known as Dengvaxia. It is a tetravalent vaccine with attenuated virus technology that uses the 17D structure of the yellow fever virus as a support, replacing the precursor membrane (prM) and envelope (E) proteins with the genes corresponding to the four dengue serotypes. It is recommended to be administered subcutaneously, with three doses given 6 months apart, to the population aged between 9 and 45 years. It is restricted to patients who have had at least one previous episode of dengue. 12 Patients who have never had contact with the virus before could develop more severe episodes of the disease on their first infection. 13

The most comprehensive study 12 on the efficacy and safety of this vaccine was carried out in 3 clinical trials with 35 thousand children aged between 2 and 16 years in Asia-Pacific and Latin America countries. This study 12 demonstrated that the overall efficacy of the vaccine against symptomatic dengue after 25 months of the full course (3 doses) was of 60.3% (95% confidence interval [95%CI]: 55.7–64.5%). The efficacy was age-dependent, with rates of 65% in children older than 9 years and of 45% in children aged ≤ 9 years. Protection for each of the virus serotypes was also different, with 40 to 50% for the DENV-1 and -2 serotypes, and 70 to 85% for DENV-3 and -4.

This vaccine also proved to be effective against hospitalization and cases of severe dengue, with 72.7% (95%CI: 62.3–80.3%) and 79.1% (95%CI: 60.0–89.0%) efficacy, regardless of serological status. Additionally, the efficacy of the vaccine in seropositive patients was 70 to 80%, while in seronegative patients it was 14.4 to 52.5%, and an increased risk of hospitalization and severe dengue fever was also observed in seronegative patients after receiving the vaccine and having a subsequent infection. 11 12 Therefore, countries that choose to use this vaccine as a strategy to combat dengue and its complications must ensure that the immunized individuals have already been infected, with serological confirmation.

The Qdenga, or TAK-003, vaccine has been approved for use in Brazil, which will be the first country to use immunization through the public health system. The priority populations are children and adolescents aged 10 to 14 years, living in cities with more than 100,000 inhabitants with a high rate of transmission of the disease in the last 10 years, also taking into account high rates of transmission in recent months. 14 The vaccine uses attenuated virus technology and covers all four serotypes of DENV. The structure of the vaccine is based on the DENV-2 PDK-53 virus and includes other chimeric DENVs replacing the *prM* and *E* genes. Its use is recommended subcutaneously, with a 3-month interval between doses.

Individuals aged 4 to 60 years can receive the immunization regardless of their dengue serological status. Its efficacy and safety were proven in a study that included more than 28 thousand participants. The individuals were observed for over 4 years and 6 months, with an overall efficacy against symptomatic dengue (virological confirmation) in the first 12 months after vaccination of 80.2% (95%CI: 73.3–85.3%). However, subsequent follow-up studies at 18, 24, and 36 months of vaccination showed a decrease in vaccine efficacy over time, with cumulative vaccine efficacy of 62% (95%CI: 56.6–66.7%). The vaccine also showed significant efficacy in preventing dengue hospitalizations: 90.4% (95%CI: 82.6–94.7%) at 18 months and 83.6% (95%CI: 76.8–88.4%) 3 years after the second dose. The decrease in efficacy over time is a target for studies to assess the possibility of including a booster dose. 11 12

The TV003/TV005 uses a technology developed by the United States National Institute of Allergy and Infectious Diseases (NIAID). It is made up of attenuated viruses. It is a tetravalent vaccine with a single dose. Its technology includes the use of recombinant DNA with deletions in the 3' untranslated region and structural genetic chimerization. This vaccine had its technology imported by Instituto Butantan in Brazil, which introduced the freeze-drying technique to circumvent the need to refrigerate the initial vaccine. Thus, a phase III clinical trial of this vaccine with 16,944 participants aged 18 to 59 years is being conducted in Brazil. 12 The first results showed an overall efficacy of 79.6% (95%CI: 70.0–86.3%) for symptomatic dengue with laboratory confirmation, 28 days after the single dose of the vaccine. Among seropositive individuals, efficacy was of 89.2% (95%CI: 77.6–95.6%), while among seronegative individuals it was of 73.5% (95%CI: 57.6–83.7%). During the study period, only DENV-1 and -2 serotypes were detected in Brazil, the vaccine showed specific efficacy of 89.5% (95%CI: 78.7–95.0%) and of 69.6% (95%CI: 50.8–81.5%) respectively. 15 As the vaccine tests have been finalized, the Butantan Institute expectation is that it will be sent still in 2024 for approval by the Brazilian Health Regulatory Agency (Agência Nacional de Vigilância Sanitária, ANVISA, in Portuguese). 16

### Vector control

Vector control brings together promising strategies for the preventive control of dengue, which aim to mitigate the transmission of the virus to humans. This control involves various mechanisms that, together, can reduce the chances of infection, including: behavioral measures, such as not leaving still water and filling plant pots with sand to avoid water accumulation; the use of chemical components, such as larvicides and insecticides, in proliferation sites, adopting focal, perifocal or spatial treatment strategies; as well as biological control, which is the most recent target of scientific research and innovation, mainly due to its ecological nature. 17

An initial population control strategy for *Ae. aegypti* is the use of spatially sprayed synthetic chemical products that aim to kill the vectors quickly. Most insecticides are of the pyrethroid chemical class, which has been the target of vector resistance by some populations. Another primary strategy is larvae control using chemical and microbial larvicides; however, its main obstacle is accessing arthropods' initial development sites. This technique is based on the biological agents of predatory copepods, fish and Toxorhynchites larvae. 18 19

There are promising alternative strategies for controlling diseases transmitted by *Ae. aegypti* , such as technologies included in the portfolio of the World Health Organization's Vector Control Advisory Group (WHO VCAG). 19

The vector contamination of *Ae. aegypti* with the entomopathogenic fungi Ascomycetes, especially *Metarhizium anisopliae* and *Beauveria bassiana* proved to be an interesting strategy for controlling the vector in larval and adult stages. In order to target and treat the habitats of larval stage mosquitoes, this technology takes advantage of self-spreading by adult mosquitoes. To contaminate the vector, it is necessary to use treated materials or dissemination stations, such as modified ovitraps. The infected mosquito dispenses the agent through contact with untreated surfaces and mating with different females. 18

Mosquito traps have proved to be an interesting vector control strategy. They use attractive signals such as light, heat, humidity, carbon dioxide, and synthetic chemicals to attract mosquitoes. An example of this technology is the Biogents Gravid Aedes Trap, which is commercially available in Brazil and has shown promising results. These traps attract and kill pregnant females, acting as lethal ovitrap. 18 19

Insecticide-treated materials (ITMs) can provide protection against bites by killing or repelling the vector. These materials have permethrin (a pyrethroid) as the active chemical component. Furthermore, ITMs can be added to clothing fabrics, ensuring protection against vector bites. However, this component does not withstand continuous washing of the fabric and degrades through constant exposure to ultraviolet (UV) rays and heat. Therefore, new formulations are being researched to achieve an effective and long-lasting release of permethrin, such as microencapsulation which binds the insecticide more deeply within the tissue. 18

The sterilization of males through irradiation and release into the environment is proving to be an interesting strategy for suppressing vector populations. The sterile insect technique (SIT) induces lethal dominant mutations in germ cells, so that mating does not produce viable offspring. The target vector's mass rearing is a major challenge for the technique's large-scale implementation, as it requires the construction of an adequate infrastructure in endemic environments. 18 20

The strategy of releasing insects carrying a dominant lethal gene (RIDL) reduces insect populations with vectorial capacity. This mechanism acts in the late larval stage and in the pupae, avoiding vector survival. It consists of generating transgenic male mosquitoes carrying a dominant lethal gene which, in association with a female-specific promoter, generate offspring composed of heterozygotes, a condition in which only males survive. Transgenics is understood to mean genetic alterations made to living organisms. This system is more advantageous than SIT, because it releases more sexually competitive insects in the wild and without a risk of radiation or release of nonirradiated animals. 18 20

The strategy of artificially infecting mosquitoes with bacteria from the Wolbachia genus is also emerging as an interesting vector control mechanism. By being present in the female germ line, the bacterium is transmitted maternally to the offspring. Artificial infection results in cytoplasmic incompatibility between infected males and uninfected females, leading to nonviable offspring. Another option for using the bacteria is its activity of inhibiting the vectorial capacity of *Ae. aegypti,* reducing virus replication and/or life expectancy. 19 21 Thus, artificially infecting the *Ae. aegypti* with this bacterium can reduce or even eliminate dengue transmission through two main mechanisms:

- Reducing the population size and/or life expectancy of mosquitoes; and
- Reducing the vector's ability to transmit the virus, since viral replication is inhibited by the presence of the bacterium in the organism. 21

A study 22 carried out in the city of Niterói, Brazil, between 2017 and 2019, demonstrated this strategy is effective in controlling the transmission of the DENV to humans. There was a 69% reduction in the number of cases compared to areas of Niterói that did not receive the technology. The Wolbachia method is cost-effective in Brazil. 23

The use of clustered regularly interspaced palindromic repeats (CRISPR)/CRISPR-associated protein 9 (Cas9) genetic manipulation technology represents hope for vector control. The transgenic element is inserted precisely at the site where it inactivates one of the sex-specific fertility genes through cleavage, thus suppressing the generation of offspring. Gene drives can also be applied as a way to replace a wild population with another, specifically resistant to the pathogen. 18 24

### Personal protection measures

Personal protection measures are those that should be widely disseminated to the population, with the aim of raising awareness about the importance of self-care in preventing dengue and its complications. These include the use of clothing that minimizes exposure and provides protection against the vector's bite, such as pants and long-sleeved shirts, the use of topical repellents composed of N-diethyl-3-methyl benzamide (DEET), icaridine or 3-[N-butyl-nacetyl] amino propionic acid, ethyl ester, (IR3535) and the installation of mosquito nets and protective structures in the home, such as window screens. 25

Topical repellents are an important strategy for preventing dengue fever and its complications. The different compositions provide different protection and safety factors. DEET is the compound most commonly found in repellents that are effective against *Ae. aegypti* . Its concentration varies from 5 to 15%, offering two hours of protection at a concentration of 5%. This product can be applied up to three times a day. Its use is only recommended from the age of 2 years. Icaridine or 2-(2-hydroxyethyl)-1 piperidine carboxylic acid, 1-methylpropyl ester is present in a concentration of 20 to 25% in topical repellents. This compound offers greater protection against *Ae. aegypti* than DEET. Its protection lasts 10 to 12 hours. Its use is considered safe for pregnant women and for children over the age of 2 years. The IR3535 compound has the lowest risk of allergy or poisoning. It provides protection for four hours and can be applied up to three times a day. It can be used from the age of 6 months. 26 27

## CONCLUSION

In conclusion, the development of vaccines, vector control through biotechnological and behavioral techniques, and personal protection measures are the most promising ways of preventing dengue and its neurological complications.

Vaccine development is a long process that requires monitoring to ensure both safety and efficacy. Takeda's Qdenga or TAK-003 vaccine is currently the most promising for application in seropositive and seronegative populations for the virus. However, other vaccines, such as TV003/TV005, whose technology has been imported by Instituto Butantan, are advanced in the testing phases, representing future hope for controlling the disease and its complications.

Vector control involves several different technologies that can help prevent the disease from spreading. To do so, these methods need to be applied in a comprehensive and sustained way, with the participation of the government and the population. It is essential to make continuous improvements to the sanitary conditions that favor vector proliferation.

Measures such as the production of specific drugs for the neurological manifestations associated with the neuroinvasive symptoms of dengue, the creation of a specific protocol for medical management of these patients, and early diagnosis in those with few or no systemic symptoms should be encouraged. All these measures are necessary to prevent and combat dengue fever and its serious and fatal complications, such as neurological symptoms.
